# Fertility preservation in women: enhanced neovascularization, follicle viability and tissue integrity in cryopreserved human ovarian cortex by remaining medulla tissue

**DOI:** 10.1186/s13048-026-02063-4

**Published:** 2026-03-16

**Authors:** Laura Rafensteiner, Sabine Eberhart, Alexandra Waldhauer, Josef Lehner, Katharina Hancke, Karin Bundschu

**Affiliations:** Department of Gynaecology and Obstetrics, University Hospital Ulm, Prittwitzstr. 43, Ulm, 89075 Germany

**Keywords:** Ovarian tissue cryopreservation (OTC), Fertility preservation, Ovarian medulla, Angiogenesis, Neovascularization, Follicle viability, Chorioallantoic membrane (CAM) assay

## Abstract

**Background:**

Ovarian tissue cryopreservation is a critical fertility preservation method for women undergoing gonadotoxic treatments. Current protocols typically discard medullary tissue, although preclinical evidence suggests the medulla may enhance neovascularization and follicle survival after transplantation. This study investigates the impact of medullary tissue retention on tissue integrity and viability in human ovarian cortex.

**Methods:**

Ovarian tissue was obtained from five women with a mean age of 27 years (20–33 years), different tumor entities (breast carcinoma [*n* = 2], B-cell lymphoma, melanoma, medulloblastoma) and AMH levels above 2 ng/mL (4.28 ng/mL ± 1.89). Cryopreserved and thawed human ovarian tissue fragments, either cortex-only, medulla-containing cortex or medulla-only, were transplanted onto the chorioallantoic membrane (CAM) of fertilized chicken eggs. Neovascularization was assessed using the CAM model by counting blood vessels converging towards the grafts and expression of angiogenic related genes (VEGFA and HIF1alpha) by qPCR. Tissue quality was evaluated by histology and immunohistochemistry for Caspase-3 (apoptosis) and Ki-67 (proliferation). Follicle viability was determined after enzymatic digestion and Calcein-AM staining.

**Results:**

Medulla-containing cortex grafts exhibited significantly higher neovascularization after grafting on the CAM compared to cortex-only grafts, with a greater number of blood vessels converging towards the graft, higher VEGFA expression and reduced HIF1A expression by qPCR measurements. The proliferation rate, measured by Ki-67 positivity, was also increased in medulla-containing cortex tissue. After CAM cultivation, the number of viable follicles was markedly greater in medulla-containing cortex than in cortex-only tissue. All tissue types demonstrated excellent post-thaw integrity and high tissue quality. Histological analysis revealed well-preserved tissue architecture and intact follicle morphology. Apoptosis was minimal, as evidenced by Caspase-3 staining with rates below 4% across all groups, indicating that the cryopreservation method for different types of tissues is unproblematic.

**Conclusion:**

This experimental study demonstrates that retaining medulla tissue in cryopreserved human ovarian cortex leads to improved neovascularization, higher cellular proliferation, reduced expression of hypoxic genes and increased follicle viability compared to cortex-only tissue after short-term CAM culture. These findings suggest that clinical protocols for ovarian tissue cryopreservation and retransplantation should consider preserving the medulla-cortex connection to optimize transplantation outcomes and functionality for fertility preservation.

## Introduction

Advancements in cancer therapy have significantly improved survival rates in recent decades [1]. Consequently, the long-term quality of life after successful treatment has become increasingly important. Young cancer patients with future reproductive intensions face the critical challenge of preserving their fertility. However, certain gonadotoxic chemotherapy and radiotherapy treatments can lead to complete ovarian failure and infertility [2–4].

Currently, the cryopreservation of ovarian cortex tissue (OTC), is the only method for fertility preservation in prepubertal girls and young women requiring urgent oncological treatment without time for hormonal stimulation and oocyte cryopreservation, or if there is a contraindication for ovarian hyperstimulation [5]. Conventional OTC involves the surgical removal, cryopreservation, and subsequent auto-transplantation of ovarian cortical tissue, which contains a high density of primordial follicles. Upon reimplantation, the tissue strips can temporarily restore both endocrine function and fertility, offering opportunity to women who might otherwise face premature ovarian insufficiency and irreversible infertility [6, 7]. To date, more than 200 live births have been reported following OTC, with reported rates of endocrine function recovery exceeding 90% and live birth rates ranging from 16 to 40%, depending on patient age and AMH level [6, 8, 9].

In addition to OTC, alternative fertility preservation strategies are under continuous development. In vitro maturation (IVM) of oocytes provides a promising option for female cancer patients who cannot undergo conventional ovarian stimulation, enabling retrieval and maturation of immature oocytes without delaying oncologic treatment [10, 11]. However, current pregnancy and live-birth rates following IVM remain lower than those achieved with standard IVF. Furthermore, in vitro growth (IVG) of ovarian follicles represents a future-oriented strategy particularly relevant for patients in whom ovarian tissue transplantation is contraindicated due to the risk of malignant cell reintroduction, such as in hematologic malignancies [12]. With advancements in three-dimensional culture models, bioreactor systems, and bioengineered matrices, IVG may eventually broaden fertility preservation options for prepubertal girls and high-risk cancer patients [13, 14]. While these techniques continue to evolve, OTC with subsequent transplantation remains the established clinical approach for prepubertal and young adult patients requiring immediate fertility preservation.

Despite these encouraging results, significant challenges remain. One of the biggest obstacles is the massive loss of follicles after transplantation. This loss is mainly caused by ischemic injury, which results from insufficient and delayed neovascularization of the grafted tissue. The ovarian cortex, when transplanted, is initially not systemically vascularized and relies on diffusion for oxygen and nutrient supply until new blood vessels form and integrate the graft [15].

Slow freezing OTC protocols involve the isolation and cryopreservation of thin strips of ovarian cortex, with the medulla discarded. Slow freezing remains the clinical standard for ovarian tissue cryopreservation, having generated the majority of reported live births to date [16]. Recent comprehensive reports from large cryobanks have provided valuable insights into storage activity and transplantation outcomes worldwide, demonstrating increasing clinical success rates [17, 18]. While vitrification has emerged as an alternative approach, showing promise in preclinical studies with recent reports of successful deliveries following transplantation of vitrified tissue [19], clinical data remain limited as tissue cryopreserved by vitrification has only recently entered the clinical process. Methodological advances, including hybrid protocols bridging slow freezing and vitrification, continue to optimize cryopreservation techniques [20]. However, recent animal studies suggest that leaving small amounts of medullary tissue attached to cortical strips may offer significant benefits. The rich vascular network and supportive stromal environment of the medulla are thought to facilitate faster and more robust neovascularization of the graft after retransplantation, thereby reducing the duration and severity of ischemia. Preclinical studies in the bovine model have demonstrated that ovarian cortex grafts containing medullary tissue improved neovascularization and follicle survival rates compared to cortex-only grafts [21].

Given the increasing demand for effective fertility preservation and the limitations of current protocols, a comprehensive evaluation of the impact of medullary tissue retention in human ovarian cortex on neovascularization and follicle viability is warranted. This study aims to address this gap by comparing the outcomes of human cortex-only and medulla-containing cortex fragments in a chorioallantoic membrane (CAM) model.

## Methods

### Ethical approval

This study was approved by the Ethics Committee of University Hospital Ulm (Approval No. 181/17). The use of ovarian tissue for patient-related research was authorized. Written, informed consent was obtained individually from each patient.

### Ovarian tissue preparation and cryopreservation

Ovarian biopsies were collected from five women (mean age: 27.2 ± 5.4 years) diagnosed with different tumor entities (breast carcinoma [*n* = 2], B-cell lymphoma, melanoma, medulloblastoma) with anti-Mullerian hormone levels above 2 ng/mL (4.28 ± 1.89 ng/mL) for regular fertility preservation treatment. Ovarian tissue was surgically removed prior to initiation of gonadotoxic cancer treatment. Immediately after removal, tissue was transferred into a tube with Custodiol^®^ (Dr. F. Köhler Chemie Pharmaceuticals, Bensheim, Germany) at 4 °C and transported to the lab for routine preparation. Tissue fragments were prepared on a cooled aluminum plate using sterile surgical blades. The ovarian cortex-medulla boundary was identified by visual inspection based on anatomical landmarks (color distinction and tissue consistency). Tissue was divided into three groups with standardized dimensions: sole cortex (5 mm x 10 mm x 2 mm, containing only cortical tissue), medulla-containing cortex (5 mm x 10 mm x 3 mm, including approximately 1 mm of medullary tissue) and sole medulla fragments (5 mm x 10 mm x 2 mm, containing only medullary tissue). Tissue dimensions were verified using graph paper. The prepared tissue fragments were incubated in OvarStore Cryo medium (basal medium: Leibovitz L15 medium + 1% HSA (Human Serum Albumin) supplemented with 6% of ethylene glycol (EG), 6% of dimethyl sulfoxide (DMSO), 0.15 M sucrose and 17 µM gentamicin sulfate) on a sample shaker at 100 rpm for 30 min at 4 °C. The slow freezing protocol was as follows: (1) starting temperature 4 °C, (2) cooling from 4 °C to -9 °C at a rate of -2 °C/min; (3) seeding at -9 °C; (4) hold at -9 °C for 10 min, (5) cooling from − 9 °C to -35 °C at a rate of -0.3 °C/min. From − 35 °C to the final storage temperature in liquid nitrogen, samples underwent uncontrolled, rapid cooling (free fall) without further programmed rate control. This protocol is based on the protocol established by Isachenko et al. (2013) [22]. Tightly closed cryovials with tissue samples were immersed and stored in liquid nitrogen, without direct exposure of tissue samples to the liquid nitrogen phase.

### Thawing

Frozen ovarian tissue was thawed at room temperature for 2 min, followed by immersion in a water bath at 37 °C for additional 2 min. Tissue fragments were washed in OvarStore Thawing medium (basal medium supplemented with 0.5 M sucrose and 17 µM gentamicin sulfate) for 15 min, followed by a gradual washing process by adding 8 mL of basal medium every 5 min over a total period of 30 min. Afterwards, tissue fragments were washed twice in basal medium and cut into 3 × 3 × 1 mm pieces for further processing. In total, out of 144 tissue fragments, 30 samples were used for follicular viability assessment after thawing, 15 samples for qPCR after thawing and 99 for grafting onto the CAM.

### Ovarian tissue grafting and CAM culture

**Fig. 1:**
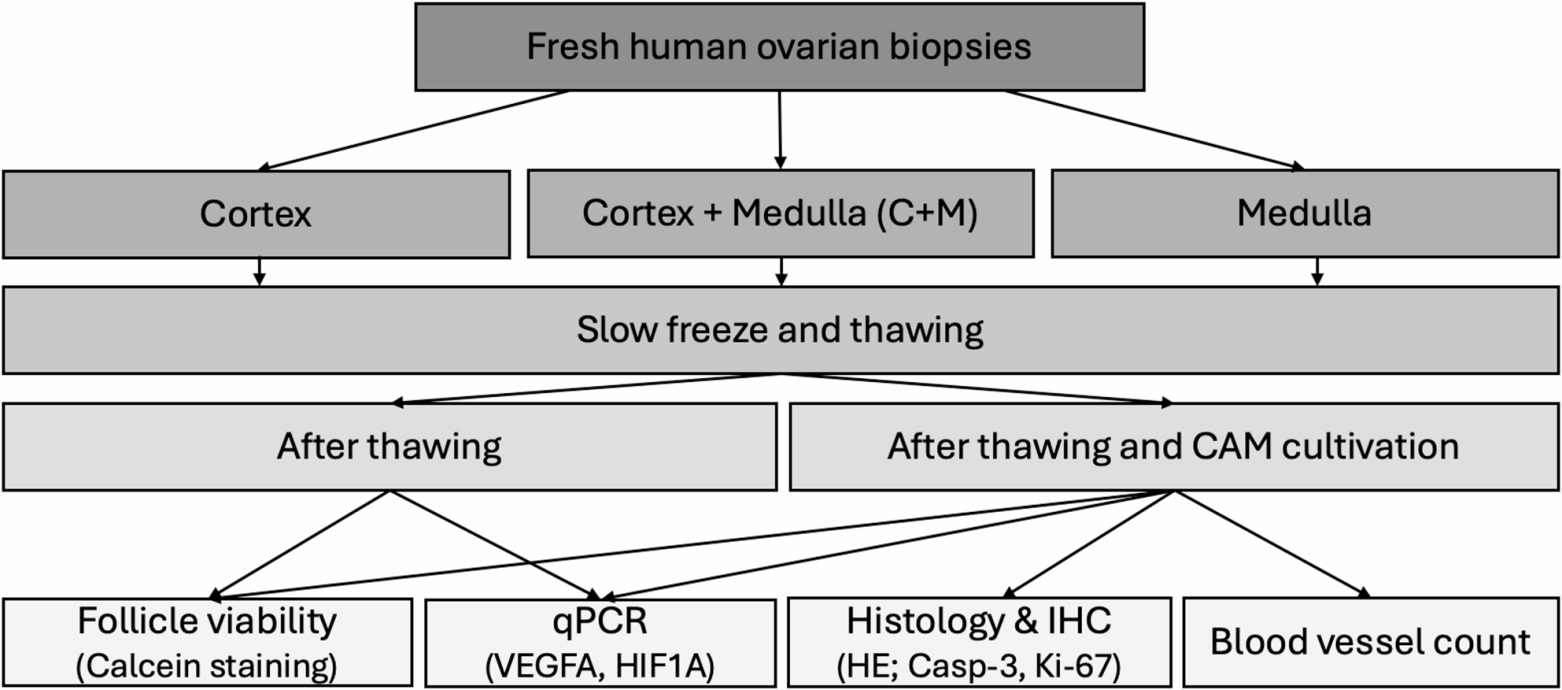
Experimental study design. Fresh human ovarian biopsies were divided into three groups (cortex, medulla-containing cortex (C + M) and medulla). Samples underwent slow freezing and thawing procedure, followed assessment either directly after thawing or after additional cultivation for 4 days on the CAM. Follicle viability was assessed by Calcein-AM staining. Molecular analysis included qPCR for VEGFA and HIF1A. Histological (HE staining) and immunohistochemical assessment (Caspase-3 and Ki-67) and blood vessel counts were additionally performed after CAM cultivation

Fertilized chicken eggs were obtained from “LSL Rhein-Main Geflügelvermehrungsbetriebe GmbH and Co KG” for tissue grafting and cultivation. Egg incubation, tissue grafting and cultivation were performed as previously described by Mueller et al. [21]. Five independent experimental series were conducted, with a total of 99 tissue samples transplanted onto fertilized eggs. On day 12 of incubation, 96 eggs remained viable, of which 30 tissue samples were used for enzymatic digestion, 15 were snap-frozen for qPCR analysis, and 54 were embedded in paraffin for histological and immunohistochemical assessments (Fig. 1).

### Determination of follicular amount and viability

Follicular counting and viability testing were performed by using Calcein-AM (Merck) immediately after thawing and following cultivation on the CAM. Pre-aliquoted Calcein-AM was dissolved in 1 x PBS (PanBiotech, Aidenbach, Germany) and combined with pre-aliquoted collagenase type 1 A (Merck) to create a working solution containing 2 µM Calcein AM and 1 mg/mL collagenase type 1 A. Tissue samples were incubated in 250 µL of working solution for 90 min at 37 °C, ensuring protection from light.

The counting of viable follicles was performed by fluorescence microscopy (Olympus, BX40) at 10 x magnification. Viable follicles were defined as those exhibiting Calcein-AM-positive fluorescence (green, 495 nm) in both the oocyte cytoplasm and the surrounding granulosa cell layer, indicating intact cellular membranes and metabolic activity. Follicles were considered non-viable if either the oocyte or granulosa cells showed incomplete or absent fluorescence [23]. Viable follicles were distinguished from individual stromal cells by their characteristic morphology (granulosa cells organized concentrically around a central oocyte) and larger size compared to dispersed stromal cells. For follicle viability assessments, follicle retention rates were calculated as percentages [(viable follicles post-CAM culture / viable follicles post-thaw) − 100%] to account for inter-individual variability in baseline follicle density and ovarian reserve (reflected by variable AMH levels: 4.28 ± 1.89 ng/mL). This approach provides a more robust comparison by normalizing for patient-specific differences in follicle distribution.

### Histology and immunohistochemistry

For histology and immunohistochemistry tissues were fixed in 4% formaldehyde solution (Otto Fischar, Saarbrücken, Germany) over night, dehydrated and embedded in paraffin. Sections of 3 μm were cut by using a microtome (RM2255, Leica Biosystems, Nussloch, Germany). Plain slides (Marienfeld, Lauda-Königshofen, Germany) were used for histology and superfrost slides (VWR International, Leuven, Belgium) were used for immunohistochemistry. Hematoxylin and eosin (HE) staining was performed in an automatic stainer (ST5020, Leica, Germany). Immunohistochemical staining was performed using Ki-67 (1:200; Invitrogen, Germany) as a proliferation marker and Caspase-3 (1:25; Invitrogen, Germany) as an apoptosis marker. Ovarian tissue sections were deparaffinized using Neo-Clear (Merck, Darmstadt, Germany), rehydrated in a series of graded ethanol baths (100%, 90% and 70%) and heat-induced epitope retrieval was performed using citrate buffer (pH 6.0) for 20 min. Endogenous peroxidase was blocked using 3% hydrogen peroxide in methanol and 5% goat serum were used to block unspecific binding. Primary antibodies were diluted in PBS and sections were incubated overnight at 4 °C in a humidity chamber. Negative control was performed in parallel, lacking the primary antibody. On the following day, sections were washed in TBS and incubated with a biotinylated secondary goat anti-rabbit antibody (1:200; Invitrogen; Germany) for 30 min in a humidity chamber at room temperature. Antibody complexes were visualized using a Streptavidin-coupled Peroxidase and DAB substrate kit (Thermo Fisher, Rockford, USA). Sections were embedded in Aquatex^®^ (Merck, Darmstadt, Germany). For Caspase-3 positive control, cryopreserved and thawed bovine medulla tissue was incubated in DMSO for 1 h and afterwards grafted onto the CAM.

ImageJ 2 (version 2.16.0/1.54p [24]) was used to quantify the area of staining in the graft, using a bright field microscope (Keyence BZ-X810). The region of interest (ROI) was manually defined around the human ovarian tissue graft (excluding CAM host tissue) and mean ratio of Ki-67/Caspase-3 per total area was reported for the different types of tissue.

To assess potential colocalization of Ki-67 and Caspase-3, immunofluorescent co-staining was performed with the CellDive Imager (Leica Microsystems) according to the standardized protocol [25]. Briefly, paraffin-embedded tissue sections (cortex, medulla-containing cortex and medulla) were deparaffinized, subjected to antigen retrieval, and blocked to prevent nonspecific bindings. Subsequently, sections were co-stained with antibodies against Ki-67 (1:800; Cell Signaling Technology) and Caspase-3 (1:100; Cell Signaling Technology). Unlabeled primary antibodies were detected using species-specific fluorescent secondary antibody (1:400; goat anti-mouse IgG Alexa Flour 488 and donkey anti-rabbit IgG Alexa Flour 647; Cell Signaling Technology).

### Quantitative polymerase chain reaction (qPCR)

Ovarian tissue samples intended for qPCR analysis were washed in phosphate-buffered saline (PBS), snap-frozen, and stored at -80 °C until further processing. Total RNA was extracted from snap-frozen ovarian tissue using the RNeasy Mini Kit (Cat. No. 74106, Qiagen) according to the manufacturer’s instructions. Briefly, tissue samples were minced with a scalpel and transferred to RLT lysis buffer containing β-mercaptoethanol and incubated on ice for 30 min. RNA purity and concentration were assessed using a Nanodrop 2000 spectrophotometer (Thermo Fisher Scientific, Germany). First-strand cDNA synthesis was performed using the QuantiTect Reverse Transcription Kit (Cat. No. 205311, Qiagen), following the manufacturer’s protocol. Quantitative real-time PCR (qRT-PCR) was conducted using the SensiFast Probe No-ROX Kit (Meridian Bioscience). Predesigned PrimePCR Probe assays (BioRad) were used to analyze the following human genes: Vascular Endothelial Growth Factor Alpha (VEGFA) and Hypoxia-Inducible Factor 1 Alpha (HIF1A). Glyceraldehyde 3-phosphate dehydrogenase (GAPDH) served as endogenous control.

### Statistical analysis

Statistical analysis was performed using descriptive statistics, with results reported as mean ± standard error of the mean (SEM).

## Results

### Good tissue quality and integrity in all types of tissue

The CAM model is an established method to cultivate different types of tissue and study short-term angiogenesis, tissue quality and ischemic processes. To determine the tissue quality and integrity of the different types of tissue (sole cortex, medulla-containing cortex and medulla-only) following transplantation and CAM cultivation, histological sections of each type of tissue were HE stained.

**Fig. 2:**
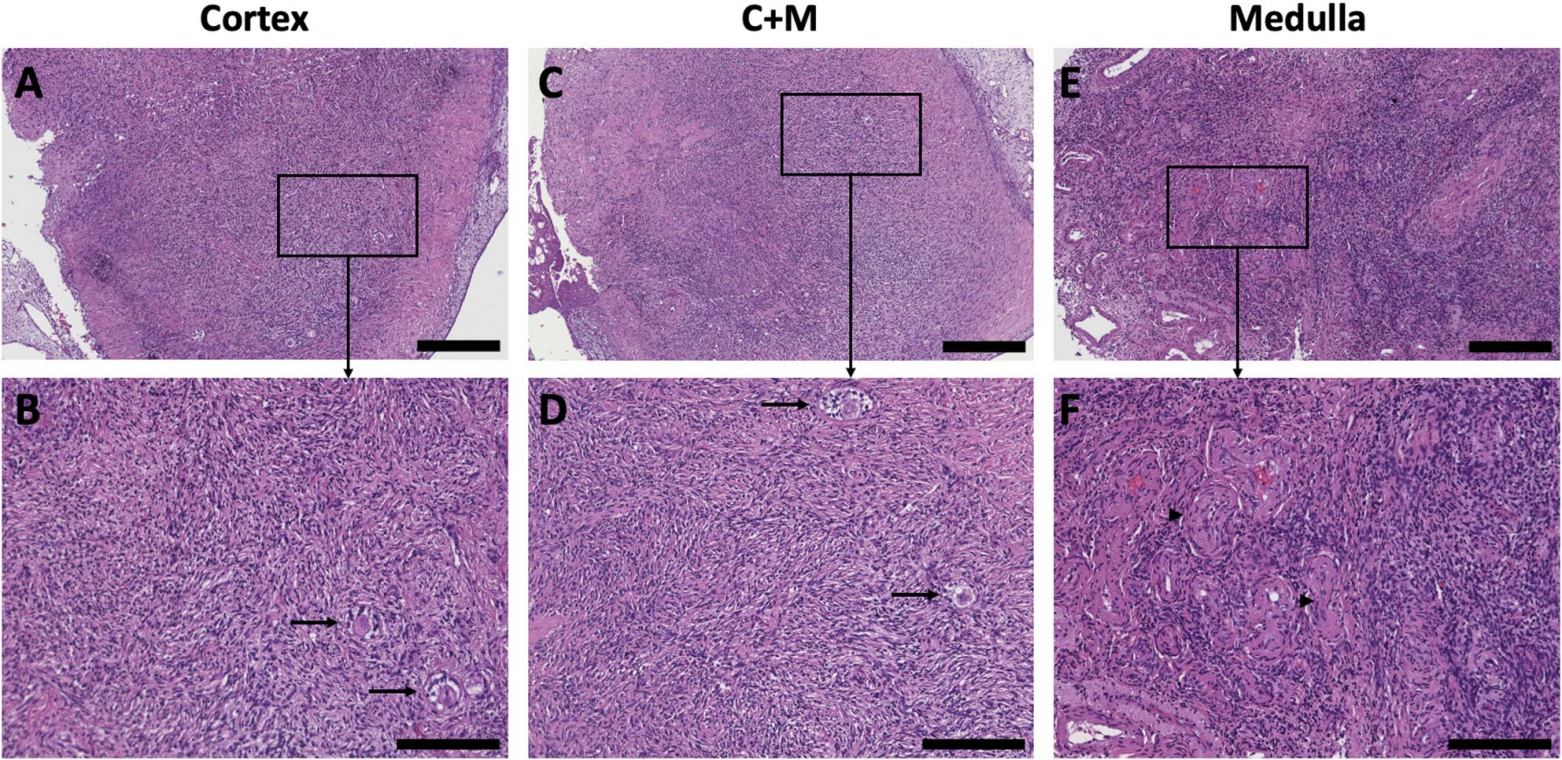
Well-preserved tissue integrity after cryopreservation and CAM culture in all tissue types. **A**-**F** Representative pictures of HE-stained tissue; **A**, **B** cortex, **C**, **D** medulla-containing cortex (C + M), **E**, **F** medulla. **B**, **D** arrows point exemplarily to follicles, **F** arrowheads point exemplarily to blood vessels. **A**, **C**, **E** Scale bar 300 μm; **B**, **D**, **F** Scale bar 150 μm

In all samples, the tissue structures remained well- preserved. Upon cultivation, only rare signs of necrosis were detected in a few of the examined ovarian tissue sections. Histological analysis using HE staining revealed well-preserved tissue architecture, with minimal evidence of necrotic features such as pyknotic, karyorrhectic or karyolitic nuclei. Additionally, no regions of tissue disintegration or loss of structural integrity were noted (Fig. 2). These observations indicate that all tissue types maintained their viability and structural integrity throughout the freezing, thawing and cultivation period.

### Higher proliferation rate in human medulla-containing cortex and low apoptosis rate in all types of tissue

Immunohistochemical analysis revealed an elevated proliferation rate in medulla-containing cortex, as indicated by a higher percentage of Ki-67-positive areas within the tissue. Specifically, the proliferating rate was increased in medulla containing cortex (31.66%), compared to cortex-only tissue (28.39%). The highest proliferation rate among of all tissue types was observed in medulla-only tissue with a value of 38.50%.

**Fig. 3:**
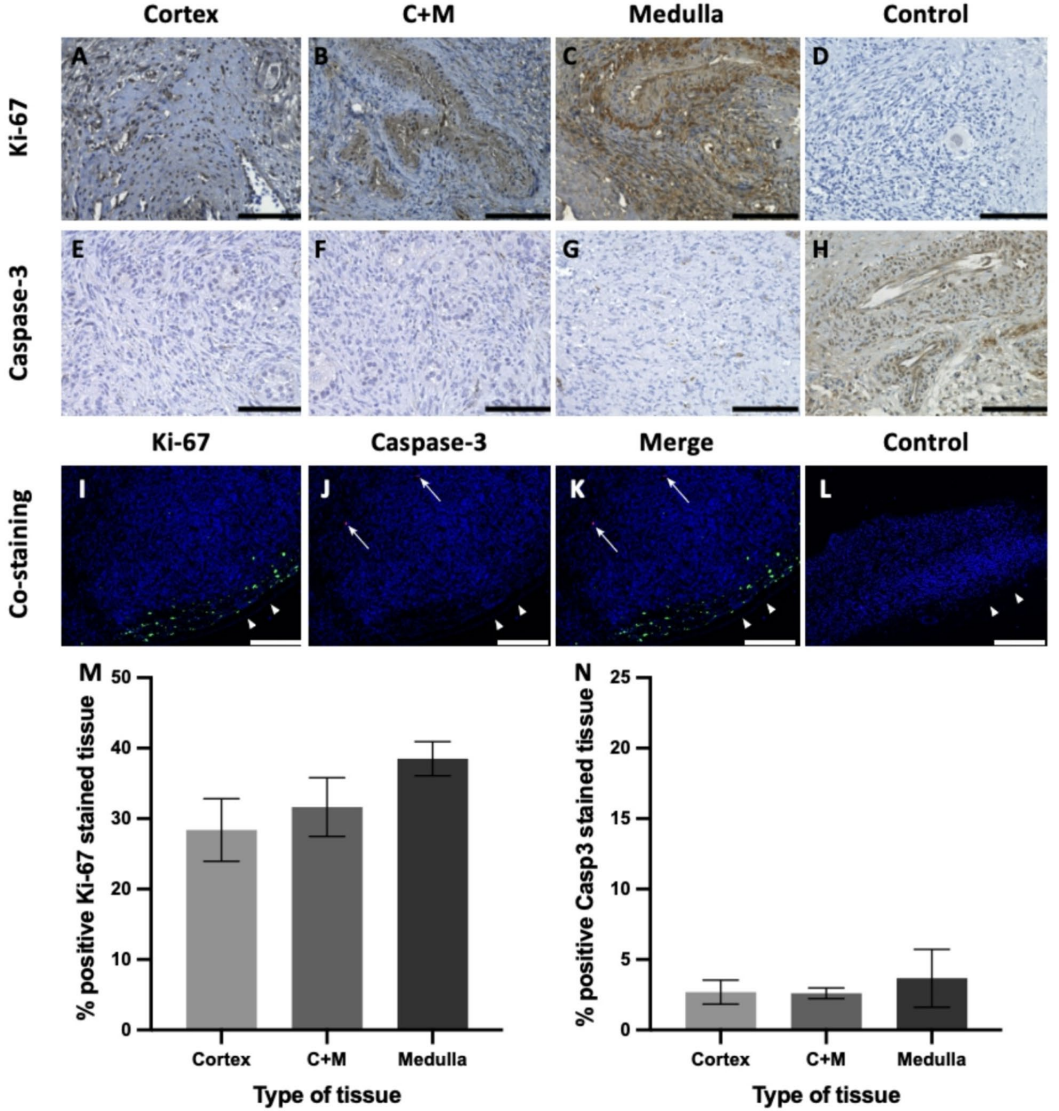
Increased proliferation and low apoptosis rate. **A**, **B**, **C** Representative pictures of Ki-67 stained (**A**) cortex; (**B**) medulla containing cortex (C + M); and (**C**) medulla tissue. **E**; **F**; **G** Representative pictures of Caspase-3 stained (**E**) cortex; (**F**) medulla containing cortex (C + M); and (**G**) medulla tissue. (**D**; **L**) Negative control lacking primary antibody; (**H**) positive control for Caspase-3 staining with DMSO treated tissue; (**I**-**K**) Representative immunofluorescence co-staining of Ki-67 (green) and Caspase-3 (red) on cortical tissue. (**I**) Ki-67 stained tissue (green); (**J**) Caspase-3 stained tissue (red); (**K**) merge of Ki-67 (green) and Caspase-3 (red) stained tissue. (**M**) Statistical analysis of % positive Ki-67-stained tissue shown by mean ± SEM; (**N**) Statistical analysis of % positive Caspase-3 (Casp3) stained tissue shown by mean ± SEM; Scale bars: 100 μm (**A**-**H**); 200 μm (**I**-**K**); Arrows pointing to Caspase 3 positive cells (red) (**J**, **K**), Arrowheads pointing to the CAM (**I**-**L**)

Following cryopreservation, thawing, transplantation and CAM cultivation, all types of tissues displayed minimal apoptosis, as indicated by rare Caspase-3 staining. Apoptosis rates were extremely low in all groups, with cortex-only tissue showing 2.69%, medulla-containing cortex 2.61%, and medulla-only 3.67% (Fig. 3). Immunofluorescence co-staining revealed a distinct spatial distribution of Ki-67 and Caspase-3 within all tissue types (cortex, medulla-containing cortex and medulla). Ki-67 expression, indicative of proliferation, was predominantly localized close to the CAM tissue. In contrast, Caspase-3 staining, indicating apoptosis, was very rare, appearing only sporadically in the central part of the tissue transplant. To integrate proliferation and apoptosis into a single metric, we calculated the Ki‑67/Caspase‑3 ratio per section. The mean ratio was almost identical in cortex-only tissue (10,3 ± 4,1), medulla-containing cortex (8,4 ± 2,8) and medulla (10,3 ± 3,7) (Fig. 3).

### Increased follicle viability in human medulla containing cortex

To further investigate the impact of ovarian medulla tissue, the number of viable follicles was examined after thawing and CAM cultivation. Tissue fragments were digested in collagenase, followed by Calcein-AM staining, and then compared between the different tissue types. The presence of dead follicles was distinguished from viable follicles based on the absence of fluorescence; only fluorescent positive follicles were counted.

Following the thawing process, the mean number of viable follicles in cortex-only and medulla containing cortex were similar. However, a clear difference was observed after tissue sample cultivation on the CAM. Following CAM cultivation, the mean number of viable follicles was 48 ± 20 in cortex-only tissue, 71 ± 20 in medulla-containing cortex, and 3 ± 1 in medulla-only tissue. However, due to the heterogeneous distribution of follicles within ovarian tissue and inherent differences in follicle density between cortical and medullary regions, direct comparison of absolute follicle numbers is limited. To account for these variations and individual differences in ovarian reserve, we calculated follicle retention rates - the percentage of viable follicles retained during CAM culture relative to the number present immediately post-thaw.

**Fig. 4:**
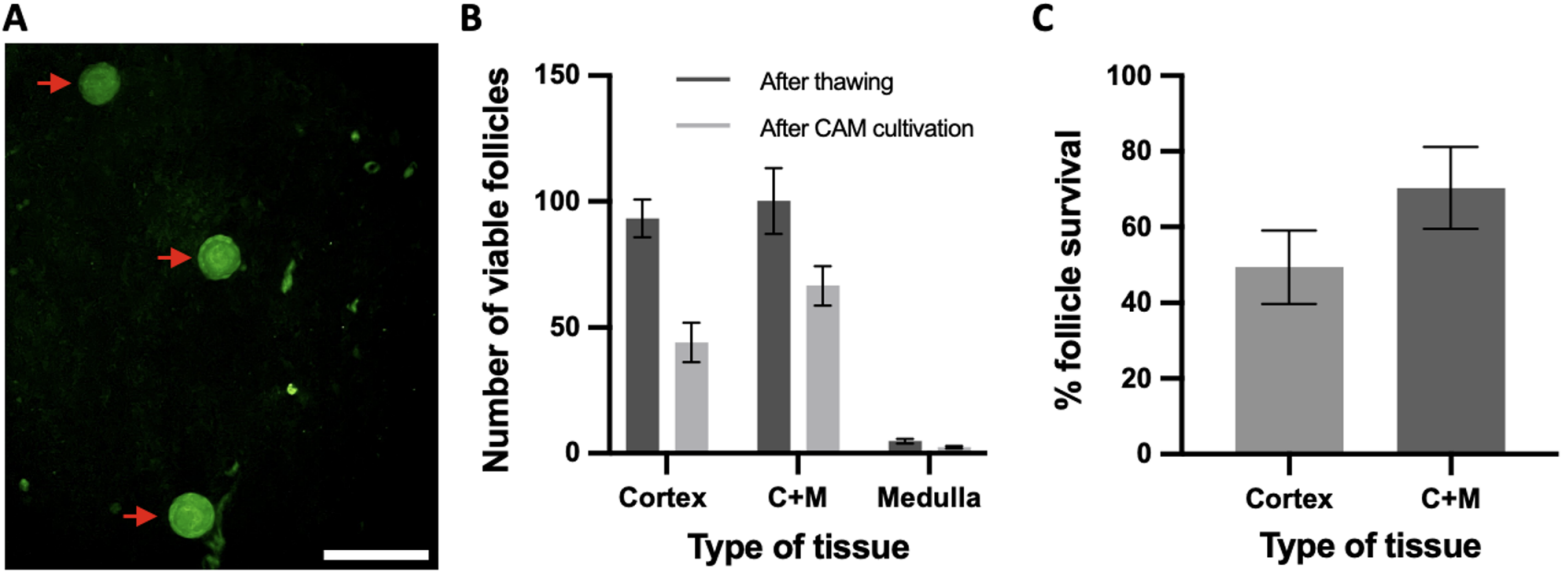
Increased number of follicle survival during CAM cultivation in the human medulla-containing ovarian cortex tissue. **A** Representative picture of Calcein-AM stained vital follicles (red arrows), scale bar 50 μm. **B** Number of viable follicles after thawing and after CAM cultivation compared in the three different tissue types presented by mean ± SEM, C + M = medulla-containing cortex. **C** Percentage of follicle survival during the CAM cultivation in relation to the previous follicle amount (after thawing) presented by mean ± SEM; C + M = medulla containing cortex

An analysis of the follicle retention per patient per tissue type demonstrated an increased number of follicles being retained in the medulla-containing cortex (70.31%) during the cultivation period compared to sole cortex (49.40%). This indicates that medulla-containing cortex preserves a higher proportion of its initial follicle population under culture conditions. As anticipated, medulla-only samples just exhibited occasional viable follicles, consistent with the known low follicle density in this region (Fig. 4).

### Increased neovascularization and reduced hypoxia in human medulla containing cortex

**Fig. 5:**
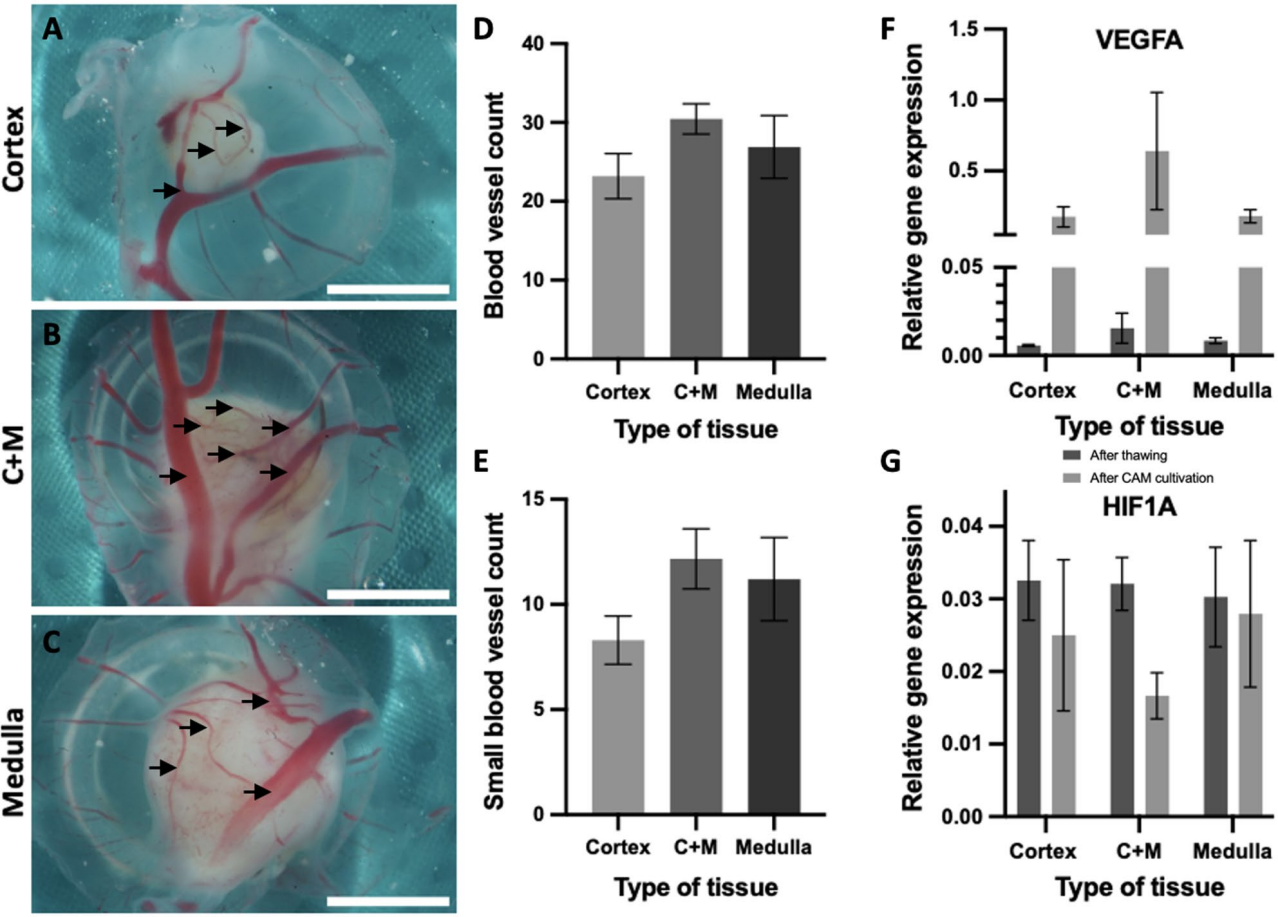
Increased number of blood vessels in medulla and medulla-containing cortex compared to sole cortex grafts. **A**, **B**, **C** Representative pictures of (**A**) cortex, (**B**) medulla containing cortex (C + M), (**C**) medulla after cultivation and harvesting of the tissue transplants; bottom views. **D** Statistical analysis of counted blood vessels is shown by mean value ± SEM. **E** Estimated number of small blood vessels is shown by mean value ± SEM; C + M = medulla containing cortex. Increased angiogenesis and reduced hypoxia in medulla containing cortex tissue compared to sole cortex and medulla tissue after CAM cultivation; (**F**) qPCR data, demonstrating relative expression of the human angiogenic gene: VEGFA; and (**G**) relative expression of the human Hypoxia-inducible factor (HIF1A); Scale bars (**A**-**C**): 3 mm

To ensure rapid oxygen and nutrient supply at the reimplanted ovarian tissue, efficient revascularization is crucial. To investigate revascularization across the three different tissue groups -cortex-only, medulla-containing cortex and medulla-only-, the CAM-model was used and all blood vessels infiltrating the tissue were quantified. Neovascularization assessments of neovascularization revealed that medulla-containing cortex grafts attracted more blood vessels compared to cortex-only grafts. Additionally, the highest number of small blood vessels was observed in medulla-containing cortex grafts, indicating enhanced angiogenic potential. Quantitative PCR analysis reinforces these findings, showing that medulla-containing cortex exhibited increased expression of angiogenic genes such as VEGFA and reduced expression of hypoxia marker HIF1A compared to cortex and medulla only tissue after CAM cultivation (Fig. 5). Given the small sample size and exploratory nature of this study, no formal statistical significance testing was performed; observed differences in gene expression patterns were consistent across all biological replicates.

## Discussion

The primary objective of this study was to investigate whether leaving medullary tissue during the routinely applied cryopreservation process of human ovarian cortex improves graft performance and thereby increases the potential for restoration of ovarian function in cases of retransplantation. Our findings demonstrate that medulla-containing cortex fragments exhibit superior tissue quality, increased neovascularization, higher cellular proliferation, reduced hypoxia and greater follicle viability compared with cortex-only grafts.

### Good tissue quality, integrity and apoptosis rates after cryopreservation and CAM cultivation of all different ovarian tissue types

HE staining confirmed well-preserved tissue architecture across all samples following slow freezing cryopreservation and CAM cultivation, supporting the applied cryopreservation protocols’ effectiveness. Despite known cryopreservation-induced stresses—such as osmotic imbalances, ice crystal formation, and oxidative damage [26, 27]—apoptosis rates remained low in all tissue types post-CAM culture. This aligns with evidence that optimized slow freezing protocols can maintain cellular integrity by minimizing cryodamage and preserving tissue morphology and function. The similar low apoptotic rates observed between medulla-containing cortex and pure cortical tissue suggest robust preservation, consistent with findings that slow freezing effectively conserves both follicular and stromal cell populations crucial for tissue viability [28]. The elevated proliferation rate in medulla-containing cortex, indicated by Ki-67 expression, confirms recent studies showing that vascularized regions benefit from improved oxygenation and nutrient supply post-thawing, enhancing regenerative potential in grafts after slow freezing [29–31].The spatial localization of Ki-67 expression to CAM-proximal regions suggests that cellular proliferation is closely linked to local tissue integration and revascularization. Given the highly neoangiogenic nature of the CAM, this pattern reflects microenvironment-dependent activation rather than unspecific or stress-induced proliferation. In addition, we calculated the Ki‑67/Caspase‑3 ratio per section as an integrated index of proliferative dominance over apoptosis. The mean ratio was very high at above 8 for all tissue types, indicating that proliferative activity clearly exceeded apoptotic cell death in all tissue types.

Currently, slow freezing remains the clinical standard for ovarian tissue cryopreservation, having generated the vast majority of more than 200 reported live births to date. This reflects both the historical adoption of slow freezing protocols and the long timeline between tissue cryopreservation and eventual transplantation. Many successful births have resulted from tissue frozen a decade or longer ago, when vitrification protocols were not yet clinically available. While vitrification has shown promise in preclinical studies for superior stromal cell preservation [28], clinical outcome data remain limited, as tissue cryopreserved by vitrification has only recently begun to be transplanted. Recent reports of successful deliveries following vitrified ovarian tissue transplantation are encouraging but represent early-stage clinical data [19]. Long-term follow-up will be necessary to fully compare the two approaches.

### Increased viable follicles in medulla-containing cortex after CAM cultivation

The follicle density in the human ovary is not evenly distributed, but exhibits marked regional heterogeneity. Primordial follicles are mainly located in the peripheral cortex, while larger, more mature follicles are localized closer to the medulla. This might be due to mechanical factors as the ovarian cortex is dense and rich in collagen, resulting in higher tissue tension, while the medulla provides a more distensible environment with reduced mechanical resistance, allowing the follicles to expand [32]. The vascularization of the medullary region supports the increased metabolic demands of the growing follicles.

Several studies have shown that follicles often occur in clusters, and their density can vary significantly within different ovarian regions [33, 34]. Female age is another crucial factor influencing follicle density and distribution. With increasing female age, both the density and total number of follicles decline markedly. Up to the age of 30, the annual follicle loss is relatively modest, however this process accelerates significantly after the age of 35, leading to a pronounced reduction in ovarian reserve [35, 36]. To account for these age-related effects, only women under the age of 35 were included. These natural variations and age-related effects were also accounted for in the quantitative analyses. The present approach evaluated the percentage of follicle retention after CAM culture relative to the number of follicles present after thawing. This provides more robust control of individual differences in baseline follicle density and age-related influences.

Confirming our hypothesis, the group with medulla-containing cortex exhibited a higher percentage of viable follicles. This may be explained by improved neovascularization and a shortened ischemic period in medulla-containing tissue, which can positively impact follicle survival after culture. These findings highlight the importance of tissue architecture and the local microenvironment for follicle viability.

### Increased neovascularization and reduced hypoxia in medulla-containing cortex

Our study provides strong evidence that ovarian tissue containing medulla exhibits enhanced vascularization compared to cortex-only grafts. This improved vascularization is crucial, as it may reduce the risk of follicle loss, which often occurs after transplantation due to ischemic injury before revascularization [37]. To prevent this massive loss of follicles, a rapid neovascularization, improved blood supply and nutrition transport in the tissue are essential. Therefore, improvement of neovascularization is highly relevant.

The use of the CAM model is an established method to study short-term angiogenesis of various graft types [38, 39]. In our experiments medulla-containing cortex had a higher number of blood vessels compared to sole cortex. This suggests that the rich vascular network and loose connective tissue of the medulla apparently form a scaffold for the rapid ingrowth of host vessels [40]. This in turn shortens the ischemia time and loss of follicles.

The mechanisms underlying the improved vascularization in medulla-containing grafts are multifactorial. First, the medulla contains pre-existing blood vessels and endothelial cells which may serve as initiation site for new vessel formation. Second, the medulla is likely to express higher levels of angiogenic factors such as VEGF, which are known to promote the development of new blood vessels. Consistent with this, our qPCR data support this hypothesis, as they demonstrate increased expression of angiogenic genes in the medulla-containing tissue.

These findings align with previous studies. Mueller et al. (2022) demonstrated in a bovine model that medulla-containing ovarian grafts, after cryopreservation and transplantation onto the CAM, exhibited a greater follicle viability and improved neovascularization compared to sole cortex grafts [21]. Kristensen et al. (2022) reported that in human ovarian grafts, revascularization was efficient from both the cortical and medullary sides, with vessel density being higher at the edges (cortex and medulla) than in the center of the graft [41]. This underscores the role of the medulla as an important vascular source and supports our findings.

Gavish et al. (2017) and other reviews emphasized that the main cause of follicle loss after transplantation is ischemic-induced activation and subsequent burnout of primordial follicles [42, 43]. Accelerated revascularization, as facilitated by medulla-containing grafts, may help to mitigate these effects. Numerous studies have also established VEGF as a key mediator of ovarian angiogenesis, essential for post-transplantation reperfusion [43–45]. Our data, showing increased expression of angiogenic genes in medulla-containing tissue, align well with these findings.

Our findings, demonstrating enhanced neovascularization in medulla-containing ovarian tissue, also align with recent research data highlighting the role of ovarian endothelial cells in promoting vascular regeneration. Spazzapan et al. (2025) showed that supplementation of human ovarian tissue with autologous ovarian endothelial cells (OVECs) significantly increased revascularization in a xenograft immunodeficient mouse model. The area of revascularized tissue in OVEC-supplemented grafts was approximately twice as large (7.14%) and with higher tissue viability, compared to not supplemented controls (3.67%) [46]. These observations strongly support the hypothesis that the human ovarian medulla, which harbors a dense network of endothelial cells, may function as an intrinsic endothelial reservoir comparable to OVEC supplementation. In this context, the medulla likely accelerates neovascularization after transplantation by providing pre-existing microvascular scaffolding and proangiogenic signals. Retaining medullary tissue during cryopreservation may therefore constitute a clinically easy and feasible strategy to improve early ovarian tissue graft revascularization by reducing ischemic injury and enhancing follicle survival.

### Clinical implications

Our study demonstrates that retaining medulla tissue during preparation, cryopreservation and retransplantation of human ovarian cortex improves neovascularization, proliferation, and follicle viability, while reducing hypoxic stress. Compared to sole cortex, medulla-containing tissue showed superior structural integrity after cryopreservation and more viable follicles post-transplantation. These findings suggest that current fertility preservation protocols should be reconsidered to include small amounts of medulla, which could be implemented with minimal adjustments to existing procedures. Future research should focus on defining the optimal amount of medulla to retain and assessing its long-term clinical relevance. Preclinical studies in mouse models are warranted to evaluate graft functionality before prospective clinical trials. Ultimately, such investigations will clarify whether improved tissue viability leads to better preservation of hormonal function, longer tissue survival, higher pregnancy rates and an increase in live births after ovarian tissue transplantation.

### Limitations

While our study provides valuable insights, it is not without limitations that deserve careful and critical consideration. First, the small sample size (*n* = 5 patients) limits statistical power and generalizability. While our results provide compelling preliminary evidence, validation in larger cohorts is necessary to confirm these findings. Second, the CAM model, while well-established for studying short-term angiogenesis, represents a xenograft system (human tissue on avian host) that does not fully replicate human vascular physiology or immune interactions. Additionally, the 4-day culture period is relatively short compared to the weeks-to-months’ timeline of clinical transplantation and graft integration, limiting our ability to assess long-term graft functionality, follicle maturation, or endocrine recovery. Also, our analyses examine only a single time point (day 4 post-grafting), time-course studies examining the kinetics of neovascularization and gene expression dynamics would provide additional mechanistic insights into the temporal patterns of graft adaptation and neovascularization. Due to the irregular geometry of the ovarian cortex-medulla boundary, precise standardization of medullary tissue inclusion was challenging, potentially introducing variability in medullary content between samples. While we used anatomical landmarks to guide tissue preparation, histological verification of tissue composition post-preparation was not determined. Our necrosis and histological assessments were primarily qualitative and descriptive, reflecting the excellent post-thaw tissue integrity observed. However, semi-quantitative analysis would strengthen the rigor of this assessment. Furthermore, histological classification of follicle developmental stages (primordial, primary, secondary) was not performed, which would further clarify which follicle populations benefit most from medullary tissue retention and whether certain developmental stages are preferentially affected by hypoxia. Future studies employing orthotopic transplantation models (e.g., in immunodeficient mice) with long-term functional assessments, including hormone secretion and follicle maturation, are warranted to bridge the gap between these preclinical findings and clinical translation.

Despite the limitations outlined above, the cryopreservation and transplantation of medulla-containing ovarian cortex may represent a valuable approach and merits further investigation and validation in larger sample sizes.

## Conclusion

In this experimental CAM-based study, human medulla-containing cortex tissue demonstrate enhanced neovascularization, increased proliferation, reduced expression of hypoxic genes and improved follicle viability compared to conventionally used cortex-only tissue. While these findings are derived from short-term preclinical experiments and do not directly assess functional transplantation outcomes in humans, they provide compelling mechanistic evidence supporting the hypothesis that retaining medullary tissue may improve graft performance. These results suggest that current cryopreservation and transplantation protocols for fertility preservation in humans should be re-evaluated in preclinical orthotopic transplantation models and, if validated, reconsidered for clinical implementation with a focus on retaining small amounts of medulla tissue attached to the ovarian cortex. Further studies are needed to determine the optimal amount of medulla to retain, assess long-term graft functionality, and evaluate clinical outcomes including endocrine function recovery, pregnancy and live births rates.

## Data Availability

Data will be made available on request.
